# Expression and Prognostic Significance of Cancer/Testis Antigens, MAGE-E1, GAGE, and SOX-6, in Glioblastoma: An Immunohistochemistry Evaluation

**DOI:** 10.30699/IJP.2020.125038.2368

**Published:** 2020-12-20

**Authors:** Seyed Abbas Tabatabaei Yazdi, Masoomeh Safaei, Mehran Gholamin, Alireza Abdollahi, Fatemeh Nili, Mehdi Jabbari Nooghabi, Kazem Anvari, Majid Mojarrad

**Affiliations:** 1 *Department of Pathology, Mashhad University of Medical Sciences, Mashhad, Iran*; 2 *Department of Pathology, Cancer Institute, Imam Khomeini Hospital Complex, Tehran University of Medical Sciences, Tehran, Iran*; 3 *Department of Laboratory Sciences, School of Paramedical Sciences, Mashhad University of Medical Sciences, Mashhad, Iran*; 4 *Department of Pathology, School of Medicine, Imam Khomeini Hospital Complex, Tehran University of Medical Sciences, Tehran, Iran*; 5 *Department of Statistics, Ferdowsi University of Mashhad,* *Mashhad, Iran*; 6 *Cancer Research Center, Faculty of Medicine, Mashhad University of Medical Sciences, Mashhad, Iran*; 7 *Department of Medical Genetics, Mashhad University of Medical Sciences, Mashhad, Iran*

**Keywords:** Glioblastoma, Cancer testis antigen, Immunohistochemistry, Prognosis

## Abstract

**Background & Objective:**

Glioblastoma is the most common primary malignancy of the brain, the prognosis of which is poor. Immunotherapy with cancer/testis (CT) antigens is a novel therapeutic approach for glioblastoma. This study aimed to investigate the expression rate of MAGE-E1, GAGE, and SOX-6 in glioblastoma tumors using the method of immunohistochemistry (IHC).

**Methods:**

Expression of MAGE-E1, GAGE, and SOX-6 were determined by IHC in 50 paraffin blocks of glioblastoma. The results were compared between variables including age, gender, tumor location, and Karnofsky performance status (Kps) score. Survival analysis was also performed.

**Results:**

The expression levels of SOX-6, MAGE-E1, and GAGE were 82%, 78%, and 76%, respectively. The relationship between CT antigens and age, gender, and tumor location was not significant, while the association between MAGE-E1 expression and age was statistically significant (*P*=0.002). High expression levels of SOX-6 and MAGE-E1 were associated with low Kps scores (*P*=0.034 and *P*<0.001, respectively). Survival analysis showed that age >40 and Kps score <80 were associated with significant relationship with shorter survival rate. (*P*=0.005 and *P*=0.018, respectively). Expression of MAGE-E1 and GAGE was negatively associated with overall 2-year survival rate (*P*=0.001 and *P*=0.021, respectively).

**Conclusion:**

The expression of all the three CT antigens, especially MAGE-E1 and SOX-6, was high in patients with glioblastoma. It can be concluded that these markers could be ideal targets for immunotherapy in such patients. MAGE-E1 and SOX-6 can be considered as important markers in determining the prognosis of glioblastoma.

## Introduction

There are 130 different types of brain tumors, among which glioblastoma is the most common. Glioblastoma comprises 15% of brain tumors (1). The incidence rate of glioblastoma increases with advancing age, with the highest incidence in individuals aged 75-84 years old (2). The average incidence rate of glioblastoma is approximately 3 per 100,000 population (3). The incidence of glioblastoma is higher in males, white race, and non-Hispanic ethnicity (4). Glioblastoma is an aggressive primary brain tumor in adults with a median survival of 15 months (5).

Despite treatment with surgery, radiotherapy, and chemotherapy, the prognosis of glioblastoma is poor (6). Age, performance status, and extent of tumor resection are the prognostic factors that are proposed as predictors of survival. However, the longest achievable survival in glioblastoma is still unclear (7). Currently, the standard treatment in patients with glioblastoma is surgical intervention, followed by local radiotherapy, as well as systemic chemotherapy with temozolomide, which is a DNA alkylating agent (8). According to the results of the European Organization for Research and Treatment of Cancer (EORTC) and the National Cancer Institute of Canada (NCIC), prospective clinical trial 26981-22981/CE.3 and surgery followed by radiotherapy with adjuvant temozolomide increased the median survival of glioblastoma patients compared to surgical treatment followed by radiotherapy alone (27% vs. 10% in 2 years) (9). 

In addition to the aforementioned treatment methods, immune gene therapy is suggested as a new method. Immune gene therapy employs different types of transport genes and has become promising in cancer treatment due to specificity in therapeutic effect based on the expressed protein(s) and low off-target effects. However, cancer cell-specific delivery of transgene(s) still poses some challenges that should be addressed (10). 

Cancer/testis (CT) antigens are protein antigens expressed exclusively in healthy adult testicular germ cells, and they are aberrantly activated and expressed in various types of human cancers (11-14). A subset of CT antigens has been found to elicit spontaneous humoral and cell-mediated immune responses in cancer patients (15). It seems that these specified antigens would be desirable targets for immunotherapy. 

Testicular cell antigens have protected immunological structure and lack human leukocyte antigen (HLA) class I; therefore, the expression of CT antigens could be immunogenic in other tissues. The rate of CT antigens expression is highly variable among different tumor types, but CT antigens are more often expressed in high-grade and late-stage cases (16, 17). Some CT antigens, including MAGE-E1, GAGE, and SOX-6 are expressed in a large number of brain tumors, which makes them potential targets for immunotherapy (18, 19). In this study, we aimed to evaluate the expression rate of MAGE-E1, SOX-6, and GAGE, as three important CT antigens, among glioblastoma patients using the immunohistochemical (IHC) technique.

## Patients and Methods

**Patient Selection**

This study was conducted on patients with pathologic diagnosis of glioblastoma who underwent a surgical operation at the Oncology Centers of Qaem and Omid Hospitals in Mashhad, Iran from 2010 to 2012.

The sample size was determined to be 50 based on the findings of the study by Lee *et al.* and using the NCSS & PASS statistical software (20).

The inclusion criteria include availability of the paraffin blocks in the Pathology Department archives and having a confirmed diagnosis of glioblastoma by a pathologist. Samples with inappropriate quality for IHC assay for each marker, sample from patients who had undergone an initial treatment before surgery, and those with incomplete clinicopathologic information were excluded from the study.

**Methods**

Demographic information of the patients, including age, gender, tumor location, and phone number were collected. Fixed paraffin blocks of each patient were sectioned and stained by hematoxylin and eosin (H & E) and were reviewed by two pathologists. Then, the proper region was marked for IHC assay; 3-4 µ sections of each paraffin block were assessed by GAGE, SOX-6, and MAGE-E1 antibodies. The applied staining kits included MAGE-E1 (ab 121161; Abcam company, US), SOX-6 (ab30455; Abcam company, US), and GAGE (h00002543-b01p; Abnova, US; containing mouse monoclonal antibody). 

The staining procedure included sectioning, preparing slides, fixation, dewaxing, antigen retrieval, endogen enzyme blocking, adding primary and secondary antibodies, stain improvement by diaminobenzidine, and hematoxylin, dehydration, and finally mounting.

The stained slides were examined by a Nikon microscope (Japan) with 100x and 400x magnification. Then, the percentage and intensity of cytoplasmic staining with MAGE-E1 and the percentage of nuclear staining with SOX6 and GAGE were evaluated and classified in all the slides.

Similar to the study by Guo *et al.* (21), samples were scored based on the percentage of the tumoral cells with MAGE-E1 positivity as follows: 0 (less than 1%), 1+ (1-10%), 2+ (11-50%), and 3+ (50%˂). We considered those scores as proportional scores.

MAGE-E1 cytoplasmic staining intensity was scored as follows: no staining (0), weak staining (1+), intermediate staining (2+), and severe staining (3+).

The final score for MAGE-E1 was calculated as combination of the intensity and proportional score as follows: negative (total score <2), 1+ (total score 2-3), 2+ (total score of 4), and 3+ (total score 5-6). A total score of negative/1+ was classified as low expression level for MAGE-E1 and a total score of 2+/3+ was classified as high expression level for MAGE-E1.

In terms of SOX-6, samples were categorized into four groups in terms of the percentage of expression of SOX-6 as follows; negative (less than 1%), 1+ or mild (1-30%), 2+ or moderate (30-70%), and 3+ or severe (more than 70%) based on the study by Ueda *et al.* (22). 

In terms of GAGE, samples were categorized into four groups including negative (less than 1%), 1+ or mild (1-10%), 2+ or moderate (11-50%), and 3+ or severe (50%) based on the study by Gjerstorff on the extent of GAGE expression in lung tumors (23). 

Based on the Kps score (24), the performance criterion of each patient was obtained and the survival rate was assessed in 24 months of follow-up. The expression levels of GAGE, SOX-6, and MAGE-E1 were compared among variables including gender, age, and location of the tumor.

**Data Analysis**

The association between Kps score and expression of the three antigens was investigated. Considering that the Kps score <80 is particularly related to a worse prognosis; patients were divided into two groups (<80 and >80) in terms of Kps score. The relationship between the Kps score and the expression of the three CT antigens was evaluated. Due to the non-normal distribution of data, based on the Kruskal-Wallis test, the expression level of CT markers was compared among gender, tumor location, and Kps score categories using the Fisher's exact test. Survival analysis was performed using the Kaplan-Meier analysis during the time interval between surgery and 24 months of follow-up, and survival curves were drawn. The relationship between survival and other variables was studied. Data analyses were performed using the statistical package for social sciences SPSS 21 (SPSS Inc., Chicago, IL., USA). A P-value of less than 0.05 was considered as statistically significant.

## Results

**Demographic Data**

Of the 50 patients, 27 (54%) were male (M:F ratio=1.1). The mean age of the patients was 43.2±12.3 years old (age range: 20-69 years old). The majority of patients (28, 56%) were in the 40-60 years group.

The most common tumor location was the frontal lobe (36%) followed by temporal lobe (26%), parietal lob (16%), occipital lobe (8%), and other areas of the brain (14%).

**Fig. 1:**
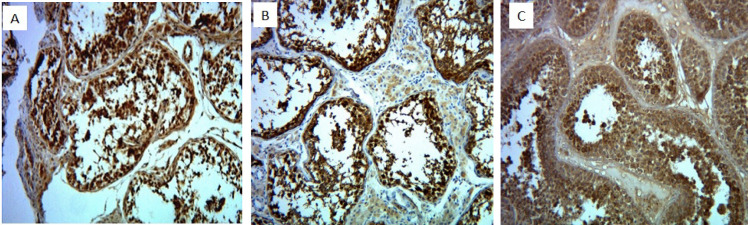
Nuclear staining of SOX10 (A), nuclear staining of GAGE (B) and cytoplasmic staining of MAGE-E1 (C) in normal testis tissue by immunohistochemistry (x100 magnification)

Staining accuracy was investigated by comparing the stained samples with human testis tissue as a positive control (Figure 1).

**MAGE-E1 Staining**

MAGE-E1 staining was negative (less than 1%) in 11 (22%) samples, +1 (1-10%) in 4 (8%) samples, +2 (11-50%) in 10 (20%) samples, and +3 (>50%) in 25 (50%) samples. Lack of staining was observed in 11 (22%) samples, while weak and intermediate staining was observed in 5 (10%) and 15 (30%) samples; severe staining was observed in 19 (38%) samples.

MAGE-E1 total scoring showed that 15 (30%) samples had low expression levels, while 35 (70%) samples obtained high expression levels.

**SOX-6 Staining**

SOX-6 staining was negative in 9 (18%) samples, mild (1+) in 11 (22%) samples, moderate (2+) in 14 (28%) samples, and severe (3+) in 16 (32%) samples.

**GAGE Staining**

GAGE staining was negative in 12 (24%) samples, mild in 26 (52%) samples, moderate in 7 (14%) samples, and severe in 5 (10%) samples.

**Fig. 2:**
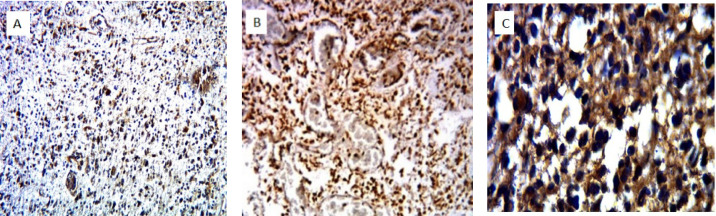
Nuclear staining for SOX10 (A), GAGE (B) and cytoplasmic staining for MAGE-E1 in glioblastoma by immunohistochemistry (A and B: x100 magnification, C: x400 magnification)

Figure 2 shows nuclear staining for SOX-6, GAGE markers, and cytoplasmic staining for the MAGE-E1 marker in glioblastoma in the present study.

**Fig. 3:**
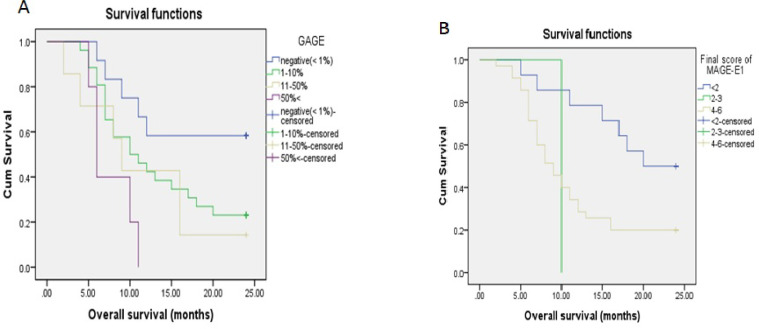
Kaplan-Meier curves showing correlation between overall survival and GAGE (A) and final score of MAGE-E1 (B). High expression l

**The Expression Level of CT Antigens and Study Variables**

***Age***

Fisher's exact test revealed a significant association between the percentage of expression and age (*P*˂0.001). This indicates that MAGE-E1 expression increased among older age groups. However, there was no significant correlation between GAGE and SOX-6 expression levels and age (*P*=0.918 and *P*=0.134, respectively) (Figure 3). 

***Gender***

The Fisher’s exact test revealed no significant association between gender and expression levels of SOX-6, GAGE, and percentage/intensity of MAGE-E1 (*P*=0.490, *P*=0.305, *P*=0.288, and *P*=0.170, respectively). 

***Tumor Location***

The Fisher’s exact test revealed no significant relationship between the location of the tumor and the expression levels of the CT antigens (*P*˃ 0.05). 

**The Relationship Between Kps Score and CT Antigens**

Among the patients, 38 (76%) had Kps score <80, and 12 (24%) obtained Kps score ≥80. There was a significant difference between the expression of SOX-6 and MAGE-E1 and also the intensity of MAGE-E1 between Kps score groups (*P*=0.034, *P*<0.001, and *P*<0.001, respectively). The increased expression levels of SOX-6 and MAGE-E1 were related to lower Kps scores. No significant difference was found in terms of GAGE expression between Kps score groups (*P*=0.149) (Figure 3). 

**Survival Analysis**

In the 24-month follow-up period of the 50 patients, 36 (72%) patients died and only 14 (28%) patients survived. The mean survival rate was 11.11 months. The overall 12-month survival rate was 46% and 24-month survival rate was 46%. A log-rank test showed a significant difference between survival and age, indicating that cases older than 40 years had shorter survival (*P*=0.005). Furthermore, a significant difference was observed in survival among patients with Kps score lower than 80 (*P*=0.018). However, the relationship between survival and gender and tumor location was not statistically significant (*P*=0.30 and *P*=0.512, respectively). 

A log-rank test showed that the GAGE and MAGE-E1 expression levels, intensity, and the final score of MAGE-E1 significantly correlated with a low survival rate (*P*=0.021, *P*=0.001, *P*=0.001, and *P*=0.037, respectively). Nonetheless, the SOX-6 expression level was not significantly associated with survival (*P*=0.325). 

**Table 1:** Expression of SOX6, GAGE and MAGE-E1 in different age groups in patients with glioblastoma

P-value	Age groups )years)						Cancer /Testis Antigens	
	60-70	50-60	40-50	30-40	20-30			
0.918	2	3	3	2	5	-/1+		**SOX6 expression**
	2	11	11	8	3	2+/3+		
0.134	4	5	10	9	7	-/1+		**GAGE expression**
	0	9	4	1	1	2+/3+		
<0.001	0	1	1	3	4	-/1+		**MAGE-E1 expression**
	4	13	13	7	4	2+/3+		

**Table 2:** Associations between SOX6, GAGE and MAGE-E1 expression and clinical characteristics in patients with glioblastoma

P-value	Tumor location					P-value	Gender			Cancer/Testis Antigens
							Female	Male		
	Other	Occipital	Parietal	Temporal	Frontal					
0.380	6	1	6	7	10	**0.490**	13	17	2+/3+	**SOX6 expression**
	1	3	2	6	8		10	10	-/1+	
0.203	3	2	0	3	4	**0.305**	3	9	2+/3+	**GAGE expression**
	4	2	8	10	14		20	18	-/1+	
0.893	6	2	4	10	13	**0.288**	13	22	2+/3+	**MAGE-E1 expression**
	1	2	4	3	5		10	5	-/1+	

**Table 3:** Associations between SOX6, GAGE and MAGE-E1 expression and Kps score in patients with glioblastoma

P-value	Kps score		Cancer /Testis Antigens	
	<80	≥ 80		
0.034	23	7	2+/3+	**SOX-6 expression**
	15	5	-/1+	
0.149	10	2	2+/3+	**GAGE expression**
	28	10	-/1+	
<0.001	32	3	2+/3+	**MAGE-E1 expression**
	6	9	-/1+	
0.001	31	3	Intermediate and strong	**MAGE-E1 intensity**
	7	9	Weak and negative	

**Table 4:** Effect of clinicopathologic parameters on the mean of survival rate in patients with glioblastoma

P-value (log-rank)	Mean and SD of survival (months)	Clinicopathologic parameters		
0.005	10.7±1.2	40<=		**Age**
	18.1±1.74	40>		
0.512	12.7±1.64	Frontal		**Tumor location**
	13.6±1.92	Temporal		
	14.8±3.24	Parietal		
	18.2±3	Occipital		
	10.14±2	Other sites		
0.30	12.3±1.4	Male		**Gender**
	14.6±1.5	Female		
0.018	18.9±1.77	80<=		**Kps score**
	11.6±1.15	80>		
0.325	18.00±2.5	-	**SOX-6 expression**	
	12.63±2.2	1+		
	12.29±1.8	2+		
	11.7±1.7	3+		
0.021	17.7±2.17	-	**GAGE expression**	
	13.7±2.17	1+		
	11.28±2.7	2+		
	7.6±1.20	3+		
0.001	18.1±2.15	-	**MAGE-E1 expression**	
	21.2±1.4	1+		
	15±2.4	2+		
	9.4±1.09	3+		
0.001	18.1±2.15	-	**MAGE-E1 intensity**	
	19±2.3	Weak (1+)		
	14±1.9	Intermediate (2+)		
	8.6±1.14	Strong (3+)		
0.037	18.6±1.7	˃2	**MAGE-E1** **final score**	
	10±0	2-3		
	11.4±1.1	4-6		

## Discussion

In the current study, the mean age of glioblastoma patients was 43 years old and 56% of the patients were in the 40-60 years age group. In the studies carried out by Johnson *et al.* and Ueda *et al.*, the mean age of the patients was 48 and 43 years old, respectively (22, 25). However, in the assessment of 449 patients with glioblastoma by Chandler *et al.*, the mean age was reported to be 39.2 years old (26), which was lower than the mean age in our study. On the other hand, Scott *et al.* reported that the mean age of the patients with glioblastoma was 53 years old, which was higher than the mean age of the patients in our study (27).

Glioblastoma is more common among males (M:F ratio=1.5 to 1); but in our study, similar to the study reported by Johnson *et al.*, the male to female ratio was almost equal to 1 (25).

In our study, the most common tumor locations were frontal and temporal lobes, which was similar to the findings of Ohgaki *et al.* (3). In contrast, Johnson *et al.* reported that the most common regions for glioblastoma were temporal and frontal lobes (25).

In the present study, MAGE-E1 positivity was observed in 78% of samples and the MAGE-E1 intensity was severe in 38% of the samples. The final score of MAGE-E1 was high (2+ and 3+) in 70% of glioblastoma cases in our study. Guo *et al.* (2003) reported an increased expression of MAGE-E1 by increasing histologic grade in glial tumors (21). Unlike some of the MAGE family members, including MAGE-A3, which do not have a high expression level in glioblastoma in some studies (28), the MAGE-E1 expression rate was high in our study. This finding was in line with the findings of the study carried out by He *et al.* (29). Therefore, MAGE-E1can be used as a target for immunotherapy.

In our study, GAGE expression was positive in 76% of glioblastoma samples, but 52% of the samples showed a mild expression level of GAGE (1-10% of tumor cells) and only 24% of the samples had an expression rate more than 10%. As a result, the expression of GAGE was somewhat different from the other two CT antigens (SOX6 expression in 60% of glioblastoma was more than 30%, and MAGE-E1 expression in 70% of tumors was more than 10%). Gjerstorff *et al.* (2006) evaluated GAGE expression in several types of cancer, including melanoma, breast, pulmonary, and liver, but not glioblastoma by polymerase chain reaction (PCR) and IHC methods. They observed heterogeneity within tumoral cells. They also observed apparent differences between GAGE antigen expression by IHC method and gene expression in some cancers; that gene expression was found higher than antigen expression (23). Furthermore, Scarcella *et al.* (1999) reported high levels of GAGE genes by RT-PCR (30). The findings of our study were in line with those of Gjerstorff *et al.* (23). Therefore, it could be hypothesized that the expression of the GAGE may vary at the cellular and gene levels in glioblastoma. Further studies are required to assess this finding. 

Correlation between the expression rates of those three CT antigens with clinicopathologic criteria in our study was as follows: MAGE-E1 expression was increased by increasing age over 40 years, and survival rate was reduced among patients older than 40 years. In contrast to the findings of our study, such association was not reported by Guo *et al.* and He *et al.* (21, 29). We could not find a relationship between the clinicopathologic criteria and level of expression of SOX-6 and GAGE. 

Our study revealed a significant correlation between high MAGE-E1 and SOX-6 level of expression and low Kps scores (Kps<80). This finding was similar to the findings of the study by Guo *et al.* (21). 

In our study, the overall 12-month and 24-month survival rates were 46% and 28% for glioblastoma patients, respectively. These findings were in line with the findings of the study by Stupp (2009), who reported a two-year survival of 27% (31). In a review by Affronti *et al.*, the survival rate for patients with glioblastoma was 69%, which was higher than the findings noted in our study, while the two-year survival rate was 29%, which was almost similar to the findings of our study (32).

Survival analysis in our study showed that aging, low Kps score, and high level of expression of MAGE-E1 and GAGE were associated with shorter survival rate. However, gender, tumor location, and SOX-6 expression had no significant association with survival rate. Correspondingly, Shinojima *et al.* (2004) reported a longer survival rate for females (33). In contrast to the findings of our study, Deltuva (2012) showed a lower survival rate for females (34).

The findings of our study revealed no relationship between the location of the tumor and prognosis. This finding was in contrast to the results of Jeremic *et al.* (1994), who reported a better prognosis for frontal lobe tumors (35).

We could not find any relationship between SOX-6 expression and survival rate. 

As previously mentioned, SOX genes can cause malignant phenotype through cell proliferation, apoptosis, invasion, and cell migration (36). We found a significant association between the high expression of SOX-6 and Kps score. Therefore, SOX-6 could be considered as an important prognostic factor for glioblastoma.

By increasing the expression of MAGE-E1 and GAGE, the overall survival rate is diminished. These two antigens play an important role in resistance to chemotherapeutic agents. MAGE-E1 reduces cell differentiation (18) and GAGE plays a fundamental role in reducing apoptosis (37). The reason for this observation might be due to the increased expression of these two markers in our study.

Generally, the survival rate of patients with glioblastoma was short in our study and the mean survival rate was less than one year (mean survival: 11.11 months). However, some previous studies reported survival rate of 20 years and more (38, 39).

Identifying the level of expression of CT antigens in patients with glioblastoma may provide a deeper insight into some novel immunotherapy treatments. Our study found high level of expression in the three CT markers, particularly MAGE-E1 and SOX-6. Based on the current and previous findings, these markers would be excellent candidates and new modalities of treatment for glioblastoma. 

## Conclusion

The level of expression of all the three CT antigens, especially MAGE-E1 and SOX-6, were high in patients with glioblastoma. It can be concluded that these markers could be ideal targets for immunotherapy in glioblastoma. MAGE-E1 and SOX-6 expression were associated with lower Kps score and lower survival rate, which indicates their possible importance in determination of prognosis in glioblastoma.
